# Ambulance clinicians’ preparedness for unplanned pre-hospital childbirth: a rapid evidence review

**DOI:** 10.29045/14784726.2025.6.10.1.47

**Published:** 2025-06-01

**Authors:** Liam Smith, Marishona Ortega, Gregory Adam Whitley

**Affiliations:** University of Lincoln ORCID iD: https://orcid.org/0009-0008-5890-0235; University of Lincoln ORCID iD: https://orcid.org/0000-0003-2647-264X; University of Lincoln; East Midlands Ambulance Service NHS Trust ORCID iD: https://orcid.org/0000-0003-2586-6815

**Keywords:** clinician, emergency, parturition, pre-hospital

## Abstract

**Introduction:**

Unplanned pre-hospital births present one of the most challenging and complex incidents for ambulance personnel to attend. This rapid review aimed to consider the barriers to providing effective maternity care in the emergency pre-hospital setting.

**Methods:**

A rapid evidence review was performed using Medline and Cumulated Index to Nursing and Allied Health Literature Complete on studies dating between 2000 and 2024. A critical appraisal and thematic synthesis were also carried out. Qualitative studies written in English that considered staff and patient perspectives for pre-hospital maternity care were eligible for inclusion in this review.

**Results:**

Six studies were identified, and three analytical themes were generated: intrinsic, extrinsic and non-technical factors impacting obstetric care. Most ambulance clinicians felt insufficiently prepared for unplanned pre-hospital childbirth. Undergraduate-level training and continuing professional development opportunities were considered poor and were acknowledged as areas requiring improvement. Academic and ambulance service organisations should work towards addressing clinicians’ lack of exposure to and confidence with maternity incidents.

**Conclusion:**

Without regulatory and organisational input, unplanned pre-hospital births will continue to challenge both newly qualified and seasoned ambulance clinicians. There is an inherent need for revised maternity training standards for pre-hospital personnel that aims to address the barriers identified within this review paper. To set forth a provision of multidisciplinary and collaborative education opportunities, pre-hospital clinicians need to be acknowledged as key care providers for pregnant women in the emergency setting.

## Introduction

In recent years, the desire to provide safe and effective maternity care has led to a stark increase in the number of childbirths taking place within a healthcare setting (McClelland et al., 2019; Strozik et al., 2023). A midwifery-led unit offers a minimal-risk environment and attempts to neutralise potential complications by providing gravid women and their babies with the appropriate level of clinical care and support (Macdonald et al., 2019; Strozik et al., 2022). Despite best efforts to safeguard this patient group, a small yet considerable proportion of births continue to occur within the community, where an emergency ambulance may be required (McClelland et al., 2019).

A birth before arrival (BBA) is one that takes place outside a suitable institution, without the support of an appropriately trained professional (Hill et al., 2022; McLelland et al., 2013, 2014). These pre-hospital births are undisputably associated with an increased risk of newborn mortality secondary to hypothermia; in BBA, babies are more likely to require neonatal intensive care admission (Loughney et al., 2006; McClelland et al., 2019; Rodie et al., 2002). Further, pre-hospital births do not avoid birthing complications such as breech, shoulder dystocia and cord prolapse (Collins et al., 2013; McClelland et al., 2019). Without access to definitive treatment and obstetric expertise, BBAs also pose a significant risk to the mother, with a death from post-partum haemorrhage occurring globally every four minutes (Loughney et al., 2006; Sebghati & Chandraharan, 2017; Strozik et al., 2022).

The incidence of pre-hospital births is ill defined, with a dearth of literature presenting variable figures across different emergency services (Heys et al., 2023). Historically, BBAs in the United Kingdom were reported at 0.5% in 2006 (Loughney et al., 2006). An upwards trend was then witnessed in a London ambulance service audit between 2007 and 2008, with a reported BBA incidence of 0.9% (Jones, 2009). More recently, the same ambulance service responded to 13,000 emergency 999 maternity incidents in 2022 (Heys et al., 2023).

The responsibility to care for these patients outside institutions inevitably lies with emergency care providers (Eisenbrey et al., 2022; Heys et al., 2023). However, the infrequency of these obstetric encounters increases the gap between learning obstetric skills and practising them, causing skill decay, with lacking confidence among ambulance personnel (Nishisaki et al., 2008; Pusic et al., 2012). As a case in point, this rapid evidence review aims to explore ambulance clinicians’ preparedness for pre-hospital childbirth and explore the impact on maternity patient experiences.

## Methods

### Study design

A rapid evidence review was performed utilising the Cochrane Rapid Review guidelines (Garritty et al., 2021). A rapid review is a time-efficient alternative to a systematic review, where evidence is gathered quickly and with less methodological rigour; this method helps fulfil the growing demand for analysis of new research by negating the need for numerous human resources and longer time frames (Garritty et al., 2021; Smela et al., 2023).

### Search strategy

To generate an answer to a clinical query, the Patient, Intervention, Comparison, Outcome (PICO) framework can be used to articulate inquiry by segmenting the question into three anatomic parts (Huang et al., 2006). The adapted PICo (population, phenomena of interest, context) mnemonic was used to produce a suitable research question with clearly defined components (Al-Jundi & Sakka, 2017; Eriksen & Frandsen, 2018). The PICo search terminology used can be seen in Table 1.

**Table 1.** PICo search terminology.

Search terms	Group		
Variable	Population	Phenomena of interest	Context
1	Ambulance*	Childbirth	Pre-hospital
2	Paramedic*	Birth	‘Out of hospital’
3	EMT		
4	Emergency medical technician		

Search string: (ambulance* OR paramedic OR EMT OR ‘emergency medical technician*’ OR MH paramedics) AND (childbirth OR birth) AND (pre-hospital OR ‘out of hospital’).

The research question being posed was: Are ambulance clinicians prepared for unplanned childbirth in the pre-hospital environment?

Databases Medline and Cumulated Index to Nursing and Allied Health Literature (CINAHL) Complete were searched via EBSCOhost for the period 2000‒2024. The Boolean operators illustrated in Table 1 were used to refine aspects of the search to ensure appropriate relevance to the research topic. An academic librarian (MO) supported the development of the search strategy.

### Inclusion/exclusion criteria

Specific inclusion and exclusion criteria were applied to ensure the reviewed literature was of high quality and specific to the research aim. These criteria are articulated in Table 2.

**Table 2.** Eligibility criteria table.

Inclusion criteria	Exclusion criteria
Published since 2000 (introduction of professional paramedic registration)	Published before 2000
English language	Not English language
Peer-reviewed academic journal	Not peer-reviewed journal
Corresponds with question/aims	Fails to address question/aims
	Full text not available

### Study screening and data extraction

All papers sourced from the search strategy were exported to Microsoft Excel for title and abstract screening. Screening was performed by LS and verified by GAW. Studies were selected that met the eligibility criteria to instigate a further full article retrieval and screening process. Full-text screening was performed by LS and verified by GAW.

Data extraction from the selected studies produced the following literature parameters: (1) author; (2) date of publication; (3) title; (4) country of study; (5) study aim; (6) participants; (7) outcome; and (8) type of study. Data extraction was performed by LS. These data were used to populate the summary of included studies shown in Table 3.

**Table 3.** Summary of included studies.

Author	Date	Title	Country	Study aim	Participants	Outcome	Type of study
Vagle et al.	July 2019	Emergency medical technicians’ experiences with unplanned births outside institutions: A qualitative interview study.	Norway	To explore emergency medical technicians’ experiences with unplanned births outside institutions.	12	Major insufficiencies exist in the knowledge and confidence of EMTs dealing with unplanned birth outside institutions.	Qualitative interview
Flanagan et al.	December 2019	Listening to women’s voices: The experience of giving birth with paramedic care in Queensland, Australia.	Australia	A narrative enquiry to identify women’s experience of unplanned out-of-hospital birth in paramedic care.	22	Maternity patients should be supported in their unplanned out-of-hospital birth process. Women’s child-birthing preferences and autonomy should always be exercised.	Qualitative interview
Hill et al.	June 2023	Paramedic training, experience, and confidence with out-of-hospital childbirth (OOHB) in Australia.	Australia	To investigate paramedic perceptions of training, confidence and experience of dealing with pre-hospital childbirth.	14	Paramedics express high anxiety and lack of confidence when attending out-of-hospital childbirth incidents. Obstetrics training is insufficient in Australia and may impact future patient care.	Qualitative interview
Persson et al.	March 2019	Specialist ambulance nurses’ experiences of births before arrival.	Sweden	To explore the experiences of specialist ambulance nurses with birth before arrival.	9	Long distances to hospital, lack of equipment and no midwifery assistance create barriers to effective maternal patient care for ambulance nurses. Scenario training is needed to increase confidence with this adversity.	Qualitative interview
Flanagan et al.	October 2019	Women’s experience of unplanned out-of-hospital birth in paramedic care.	Australia	A narrative enquiry to identify concerns with paramedic practice from the patient’s perspective.	22	Several factors, such as privacy, interpersonal skill and consent, were highlighted as improvement areas for paramedics.	Qualitative interview
Svedberg et al.	July 2020	Women’s experiences of unplanned pre-hospital births: A pilot study.	Sweden	To describe the care provided to pregnant women by emergency care clinicians.	8	Pre-hospital birth is described as a tumultuous event. Ambulance nurses should remain calm and exercise safety. Technical skills can also be improved to advocate for the mother and her wishes.	Qualitative questionnaire

### Critical appraisal

Critical appraisal is the research skill of allocating value and relevance to clinical research and dividing high-quality evidence from low-quality data (Al-Jundi & Sakka, 2017; Gajbhiye et al., 2021). The qualitative conditions of this review required a corresponding critical appraisal methodology, for which the Critical Appraisal Skills Programme (CASP, 2018) qualitative checklist was used. Each paper was screened to answer questions 1‒10. The results of these findings can be found in Supplementary 1. Critical appraisal was performed by LS and verified by GAW.

### Synthesis technique

Thematic synthesis derived from Thomas and Harden (2008) was performed to synthesise key themes from the included qualitative review papers. This process uses three stages to produce hypotheses that can subsequently be tested against quantitative research:

1. Quotation and text coding.
2. Descriptive theme development.
3. Analytical theme generation.

Synthesis methods were performed in line with the allocated time and resources available. Pertinent adaptations included developing codes from data extraction and creating key themes by hand. Thematic synthesis was performed by LS manually, without the use of software.

## Results

**Figure 1:**
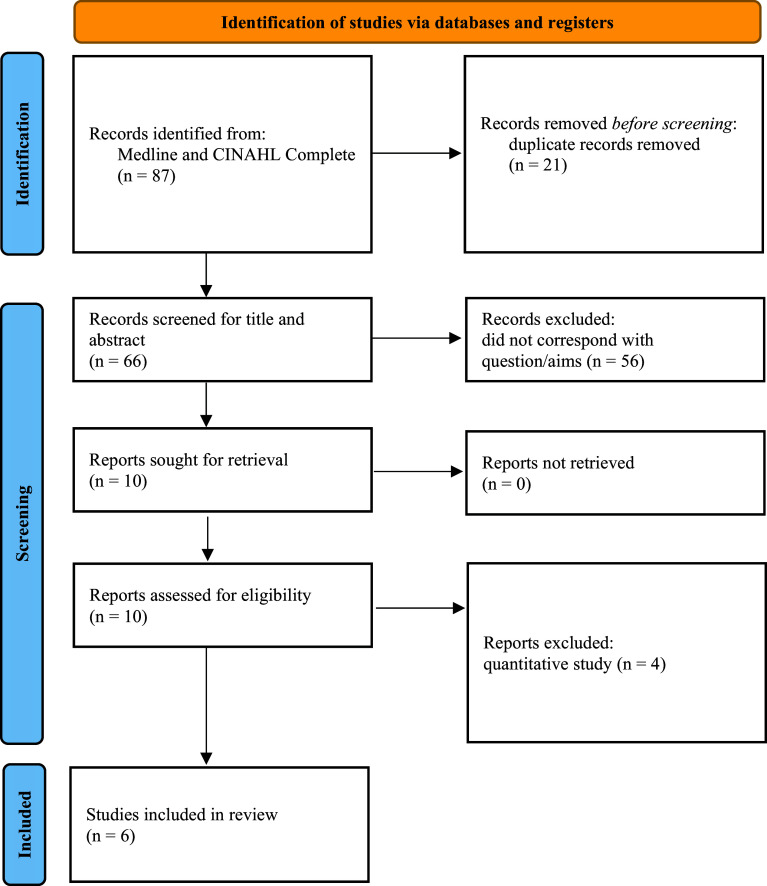
Figure 1. PRISMA flow diagram.

The Medline and CINAHL Complete database search strategy produced 87 papers. After duplicate removal, 66 titles remained for title and abstract screening, of which only 10 were relevant and sought for full-text retrieval. These 10 papers were assessed for relevance against the review question and four papers were excluded from the review due to their quantitative methodology. The six final studies met the eligibility criteria and were included in the rapid evidence review. Figure 1 presents the preferred reporting items for systematic reviews and meta-analyses (PRISMA) flow-chart results.

### Critical appraisal results

Included papers were assessed for risk of bias using the CASP qualitative checklist. Four studies failed to adequately acknowledge the relationship between the researcher and participants. One paper had a risk of researcher bias in its analysis section with unclear data. These critical appraisal findings impair the researcher’s ability to confidently answer the research question. A comprehensive table of critical appraisal findings can be found in Supplementary 1.

### Synthesis

**Figure 2:**
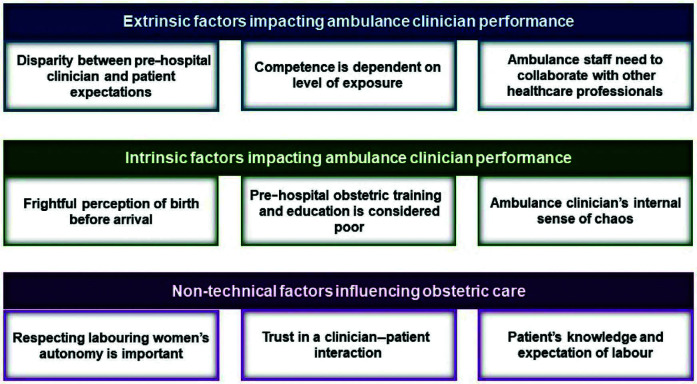
Figure 2. Thematic map.

Six papers were included in the qualitative synthesis, interviewing emergency medical technicians (Vagle et al., 2019), paramedics (Hill et al., 2023), specialist ambulance nurses (Persson et al., 2019) and birthing patients (Flanagan et al., 2019a; Flanagan et al., 2019b; Svedberg et al., 2020). Three analytical themes were generated using the thematic synthesis technique as described by Thomas and Harden (2008): extrinsic factors impacting ambulance clinicians’ performance, intrinsic factors impacting ambulance clinicians’ performance and non-technical factors influencing obstetric care. The three analytical themes were derived from nine descriptive themes and 32 initial codes (see Supplementary 2 for the complete thematic synthesis table). The thematic map shown in Figure 2 illustrates these themes and subsequent sub-themes. All supporting quotations used throughout the synthesis can be found in Supplementary 2.

#### Extrinsic factors impacting ambulance clinician performance

Ambulance clinicians felt unsupported in their experiences of communicating with other healthcare professionals (see quotations 1‒4). This lack of support came from numerous midwives and a doctor on one account (Persson et al., 2019; Vagle et al., 2019). This negated a trusting interprofessional relationship, which hindered the paramedics’ ability to access support from other professionals in the community (Persson et al., 2019; Vagle et al., 2019).

Ambulance staff perceived their patients’ expectations as high, despite being taught very little about obstetric care (see quotations 5‒7). On the contrary, Hill et al. (2023) reported an example of disengaged paramedics during obstetric training sessions, which impedes their ability to care for labouring women (see quotation 8). Flanagan et al. (2019b) found that conflict arose when a patient’s maternity expectations of healthcare providers were unmet.

The prior experiences of ambulance clinicians seemed to influence their perceived competence and confidence in treating both obstetric and newborn patients. Many clinicians described their unsubstantial level of exposure with childbirth incidents (see quotations 9‒10). In opposition, some clinicians explained that their confidence had grown after attending more maternity incidents, where they could derive patterns from previous experience (Hill et al., 2023; Vagle et al., 2019). Many ambulance clinicians with little experience of pre-hospital childbirth suffered skill fade due to not having the opportunity to practise their clinical skills (see quotations 16‒18; Hill et al., 2023).

#### Intrinsic factors impacting ambulance clinician performance

Another established theme was that pre-hospital maternity training and education are considered poor. Many emergency workers expressed the need for simulation drills and practice placements to hone their clinical skills (see quotations 19‒21; Hill et al., 2023; Vagle et al., 2019). One participant alluded to the inconsistency of clinical placements; not all student clinicians are guaranteed experience on a neonatal care unit or delivery suite (see quotation 21; Vagle et al., 2019). Patient experiences also acknowledge incompetent staff with a lack of experience, which created anxiety for the labouring mother (see quotations 22‒23; Flanagan et al., 2019a). Differing local guidelines between ambulance trusts added to the uncertainty among ambulance staff and their permitted scope of practice (see quotation 24; Hill et al., 2023).

Many ambulance clinicians experienced an internal sense of chaos and stress when attending labouring women (see quotations 25‒31). Birthing patients express gratitude for paramedics who make them feel at ease, whereas paramedics find keeping calm in a stressful childbirth situation challenging (Persson et al., 2019; Svedberg et al., 2020; Vagle et al., 2019). One account acknowledged the difficulty behind managing the complications of two patients by themselves (Persson et al., 2019).

The unpredictability of labour was a common theme among many staff, which created a perception of birth before arrival being frightful (see quotations 32‒40; Hill et al., 2023; Persson et al., 2019; Svedberg et al., 2020). Some also agreed that maternity incidents can be complex, as there can be difficult decisions to make and many tasks to focus on (Hill et al., 2023; Persson et al., 2019). However, one contrasting view was for emergency workers to perceive birthing women as healthy people undergoing a natural process (Hill et al., 2023).

#### Non-technical factors influencing obstetric care

A major consensus was reached on building trust in a clinician‒patient interaction (see quotations 41‒60). Both patients and ambulance clinicians presented positive outcomes when effective communication was used during maternity incidents (Flanagan et al., 2019a; Hill et al., 2023; Svedberg et al., 2020). Poor efforts at communicating and a lack of empathy created antithetical results, where labouring women did not feel supported in their birth experience (Flanagan et al., 2019a; Hill et al., 2023). There were a small number of unfortunate issues with informed consent and unprofessional conduct, where paramedics did not always act professionally in the best interests of their patients (see quotations 54‒60; Flanagan et al., 2019a); these experiences led to impaired levels of trust within patient‒clinician interactions.

Multiple sources described the importance of respecting labouring women’s autonomy (see quotations 61‒80). Flanagan et al. (2019b) found that many patients favour the natural birthing process, and some are prepared to decline medical interventions. These subjective standpoints had implications for building rapport in some patient‒clinician interactions, as paramedics are taught and advocate for the clinical management of birth (Flanagan et al., 2019b). Women placed trust in ambulance staff in situations where labouring women were made to feel safe and supported (Svedberg et al., 2020).

Svedberg et al. (2020) and Flanagan et al. (2019b) reported that patients’ knowledge and expectation of labour are shaped by existing birth knowledge (see quotations 81‒87). Some gravid women trusted their instincts and were surprised by how the body adapts to the child-bearing process (Flanagan et al., 2019b; Svedberg et al., 2020). Many pregnant women share the same viewpoint as ambulance clinicians in that birth is an unpredictable event (Flanagan et al., 2019b; Svedberg et al., 2020).

## Discussion

This rapid review discovered that most ambulance clinicians are ill-prepared for unplanned pre-hospital childbirth. Several intrinsic and extrinsic factors impede their ability to manage obstetric incidents in a calm and organised manner, which results in anxiety among labouring women and untrustworthy interactions. Patient experiences have highlighted the need for further training on both technical and non-technical skills.

The need for trust in a patient‒clinician interaction is consistent in the context of planned out-of-hospital childbirths and complements this review’s findings (Lang et al., 2021). A randomised controlled trial by McLachlan et al. (2015) explored midwifery care for planned community homebirths; they found that labouring women emphasise the need for a trusting relationship with their care provider. Similar studies report that pregnant women prefer the continuity of a midwife throughout the antenatal and labouring process (Boucher et al., 2010; Lang et al., 2021). Baranowska et al. (2021) found that seasoned midwives use their intuition to adopt various communication techniques that put labouring women at ease, such as eye contact and therapeutic touch. This places ambulance clinicians in a disadvantaged position, as they naturally lack the underpinning clinician‒patient trust from previous clinical encounters and are not seasoned in maternity care (McLachlan et al., 2015; Tikkanen & Sundberg, 2023). Although complementary to this review’s findings surrounding trust, the lack of these techniques presents new barriers to effective pre-hospital care delivered by ambulance staff. To balance the trust differences between planned and unplanned settings, ambulance clinicians should feel comfortable with the clinical basics of maternity care to facilitate the delivery of non-technical skills.

There are strong links between the unpredictability of planned out-of-hospital births attended by midwives and unplanned births attended by ambulance clinicians (Flanagan et al., 2019b; Lang et al., 2021). Due to the unchangeable volatility of childbirth, urgent transfer to hospital may arise even in ‘planned’ childbirth despite antenatal screening for risk factors (Svedberg et al., 2020). The likelihood of hospital conveyance in the UK is as much as 45% for women birthing their first child, or nulliparous women (Brocklehurst et al., 2011). A retrospective analysis by Strozik et al. (2023) argues that complications still occur despite the presence of highly skilled and experienced midwives; the most common indications for emergency transport were retained placenta and postpartum haemorrhage. These factors are circumstantial and complement this review’s extrinsic factor findings that are outside the control of the clinician. It is undeniable that obstetric emergencies are challenging for both midwives and paramedics due to lack of resources and medical equipment, which should be considered throughout homebirth planning.

The need for creating strong interprofessional relations is acknowledged from both the pre-hospital clinician and midwife perspectives (McLelland et al. 2016; Persson et al., 2019; Vagle et al., 2019). McLelland et al. (2016) attribute this to poor previous experiences between midwives and ambulance workers in hospital and community settings, whereas Cox et al. (2013) argue this issue is not widespread, as some midwives and paramedics have good professional relations. To mitigate interprofessional conflict, simulation-based training involving both parties has been found to improve patient outcomes by creating established relationships and shared understanding (McLelland et al., 2016; Newman, 2022). A national drive for interdisciplinary training has recently been recommended to improve collaborative working, but it is yet to be implemented across higher education (Heys et al., 2022; NHS England, 2019).

### Strengths and limitations

These findings have highlighted the lack of qualitative research surrounding pre-hospital obstetric care and could form scaffolding for future research. To complement the international endeavour of medical study, papers were gathered from across the world to produce balanced results from different ambulance services; these papers also had a low risk of bias, which adds to the validity of the review’s outcomes.

This rapid review was not exempt from limitations. First, there is no global standardisation for the role of ‘paramedic’, hence the use of ‘ambulance clinicians’ in this research enquiry. Some countries use the term ‘ambulance nurses’, while others use ‘emergency medical technicians’, which creates difficulties in drawing generalised inferences between sampled populations. Second, the streamlined search strategy using only one researcher and two databases creates bias, as pertinent studies may have been missed. Third, only articles written in English were included, which creates language bias and may have excluded relevant papers written in other languages, thereby reducing the validity of this review’s findings. Fourth, due to limited time and resources, we were unable to fully assess the strengths and limitations at study and outcome level. Finally, this qualitative review is hypothesis-generating and cannot make statistical inferences in the absence of numerical data.

### Implications for clinical practice and future research

Quality improvement for clinical practice should focus on delivering standardised models of collaborative and interdisciplinary obstetric training for pre-hospital clinicians, through undergraduate education or continuing professional development opportunities. Educational institutions and ambulance services should work to address the barriers to good patient outcomes highlighted in this review and to reduce the culture of fear surrounding obstetric emergencies.

Further research into the effectiveness of paramedic interventions would present specific developmental areas regarding the pre-hospital maternity scope of practice. More out-of-hospital research is needed in other developed countries, such as the UK, USA and Canada, to better understand patient and professional experiences across the globe.

## Conclusion

Pre-hospital maternity care needs attention. This rapid review presents the distinct influence of ambulance clinicians on women’s pre-hospital birth experiences. The imperishable and unpredictable nature of unplanned childbirth will continue to challenge emergency practitioners on an international level without regulatory and organisational input. Further research into potential improvements for paramedic education and pre-hospital maternity care could help to improve clinical competence.

## Author contributions

LS was responsible for conceptualisation, methodology, formal analysis and writing of the original draft. MO was responsible for methodology and resources. GAW was responsible for methodology, writing (review and editing) and supervision. LS acts as the guarantor for this article.

## Conflict of interest

None declared.

## Ethics

Not required.

## Funding

None.
